# The Putative Antidiabetic Effect of *Hypericum perforatum* on Diabetes Mellitus

**DOI:** 10.3390/ijms26010354

**Published:** 2025-01-03

**Authors:** Aikaterini Theodorakopoulou, Ioanna Pylarinou, Ioanna A. Anastasiou, Nikolaos Tentolouris

**Affiliations:** 1 Diabetes Center, First Department of Propaedeutic Internal Medicine, Medical School, National and Kapodistrian University of Athens, Laiko General Hospital, 17 Agiou Thoma Street, 11527 Athens, Greecepilarinoujoanna@gmail.com (I.P.); anastasiouiwanna@gmail.com (I.A.A.); 2 Department of Pharmacology, Medical School, National and Kapodistrian University of Athens, 11527 Athens, Greece

**Keywords:** diabetes mellitus, St. John’s Wort, inflammation pathway, hypericin, hyperforin, in vitro studies, in vivo studies, oxidative stress

## Abstract

Diabetes mellitus (DM), a global disease that significantly impacts public health, has become increasingly common over time. In this review, we aim to determine the potential benefits of St. John’s Wort (SJW) as an adjunct therapy for DM. We gathered information from studies conducted in vitro, in vivo, and in humans. In vitro studies investigated the concentrations of SJW extracts capable of inhibiting certain enzymes or factors involved in the inflammatory pathway, such as the β-signal transducer and activator of transcription 1, nuclear factor κB, methylglyoxal, and oxidative stress (OS). The extract was found to have positive effects on OS and anti-inflammatory properties in DM, suggesting it could serve as a protective agent against diabetic vascular complications, cell damage, and apoptosis. According to in vivo research, the essential components of the extract can stimulate thermogenesis in adipose tissue, inhibit several key inflammatory signaling pathways, and delay the early death of pancreatic β cells, all of which contribute to combating obesity. The extract may also help treat prediabetes and significantly reduce neuropathic pain. Human studies have also confirmed some of these results. However, some of the plant’s side effects need further investigation through clinical research before it can be used to treat DM.

## 1. Introduction

Diabetes mellitus (DM) is a global disease with a significant public health impact and a steadily increasing prevalence over the years [1]. The latest data show that approximately 537 million adults worldwide have DM, and this number is expected to increase by 46% to 784 million people by 2045. It is estimated that nearly 6.7 million deaths worldwide were caused by DM in 2021, equivalent to one death every 5 s [1].

According to the American Diabetes Association, DM is a heterogeneous metabolic disorder characterized by chronic hyperglycemia [2]. It results from abnormal insulin secretion or activity, or a combination of both, and is responsible for serious macrovascular and microvascular complications such as cardiovascular disease (CVD), diabetic nephropathy, peripheral and autonomic neuropathy, and diabetic retinopathy. Type 2 DM (T2DM) is the result of resistance to the action of insulin and a progressive decrease in its secretion by the β cells of the pancreas, while type 1 DM (T1DM) is caused by autoimmune destruction of the β cells of the pancreas, leading to a complete lack of insulin. Apart from these two large and most numerous categories, there are many other types of DM that arise from various causes, such as various syndromes and diseases, medications and chemical substances, etc. [2].

Today, the mechanism by which hyperglycemia occurs and causes all these adverse complications to the human body is not fully understood [3]. The resulting intense metabolic stress is thought to be responsible for inducing chronic low-grade inflammation, which is linked to obesity, atherogenesis, and diabetes [3,4,5]. Numerous studies over the years have confirmed the association between DM progression and inflammation, which is caused by increased levels of pro-inflammatory cytokines and chemokines and is responsible for various histological changes in the pancreatic islets of individuals with T2DM, such as immune cell infiltration, amyloid deposition, cell death, and fibrosis. Inflammation is an evolutionarily selected protective response of the immune system against harmful stimuli, which acts by removing noxious stimuli and external stressors, thus contributing to the restoration of tissue homeostasis and resolution of acute symptoms. However, uncontrolled acute inflammation can become chronic, which, in turn, can lead to a large number of chronic inflammatory diseases [3,4,5]. Current research on the ability of SJW to inhibit the synthesis of advanced end glycation end products (AGEs), its effects on wound healing, and its ability to relieve neuropathic pain were included in this review.

It is imperative to find complementary therapies that will simultaneously target the underlying disease mechanisms, which include inflammation [6]. One class of such treatments could be plant extracts, which already seem to hold an important place in the alternative treatment of many different diseases. For example, dietary polyphenols, which are mainly found in fruits, vegetables, tea, coffee, chocolate, etc., stand out for their antioxidant and anti-inflammatory actions and, by extension, for protection against chronic, autoimmune diseases, including DM, where they are thought to improve insulin secretion and reduce insulin resistance [6].

In the present review, we gathered data from in vitro, in vivo, and in-human studies, indicating a positive association between St. John’s Wort or *Hypericum perforatum* L. (SJW) as an alternative or complementary treatment for DM. The flavonoids of the extract are related to its antioxidant and neuroprotective action [7,8]. Today, SJW is primarily known for its use against depression, anxiety, and sleep disorders, making it widely used, with the highest sales occurring in Germany. The association of the plant with depression-related mechanisms has been based on the strong affinity hypericin (HYP) appears to have for dopamine receptors. Another potent compound, hyperforin (HPF), appears to exert antibacterial activity by inhibiting the growth of microorganisms such as Gram-negative bacteria and *Staphylococcus aureus*. HPF is considered a lead substance with diverse pharmacological properties, such as antidiabetic, antitumor, antidepressant, and antidementia-related effects [9,10]. Researchers have also found that HPF inhibited IL-13- induced nasal epithelial cell inflammation and barrier damage by targeting B-cell lymphoma 6 (BCL6), p38 mitogen-activated protein kinase (MAPK), and C motif chemokine 11 (CCL11). This finding is particularly interesting, as it could offer promising therapeutic targets for allergic rhinitis [11].

## 2. Selection Criteria and Search Strategy

Although this manuscript is not a systematic review, we conducted a literature search for its preparation using MEDLINE, EMBASE, and the Cochrane Library, focusing on English-language publications published between 2011 and October 2024. The terms included ‘hypericin’, ’hyperforin’, ‘diabetes mellitus’, ‘St. John’s Wort’, and ‘treatment’, both individually and in combination with the search strategy.

Our analysis of the published literature included data from in vitro, in vivo, and human studies, which indicate a positive association between SJW and its potential use as an alternative or complementary treatment for DM.

## 3. St. John’s Wort

Hypericum is one of the oldest species of medicinal plants, first described in ancient Greece by Hippocrates [7]. There are various species and strains of the plant, each offering different therapeutic effects, with the best-known being antipyretic, antiviral, antibacterial, diuretic, analgesic, antioxidant, anti-inflammatory, and antidepressant properties. Moreover, in traditional Chinese medicine, Hypericum has also been used to eliminate toxins, treat hemoptysis and hematemesis, and relieve muscle pain [7].

**Figure 1:**
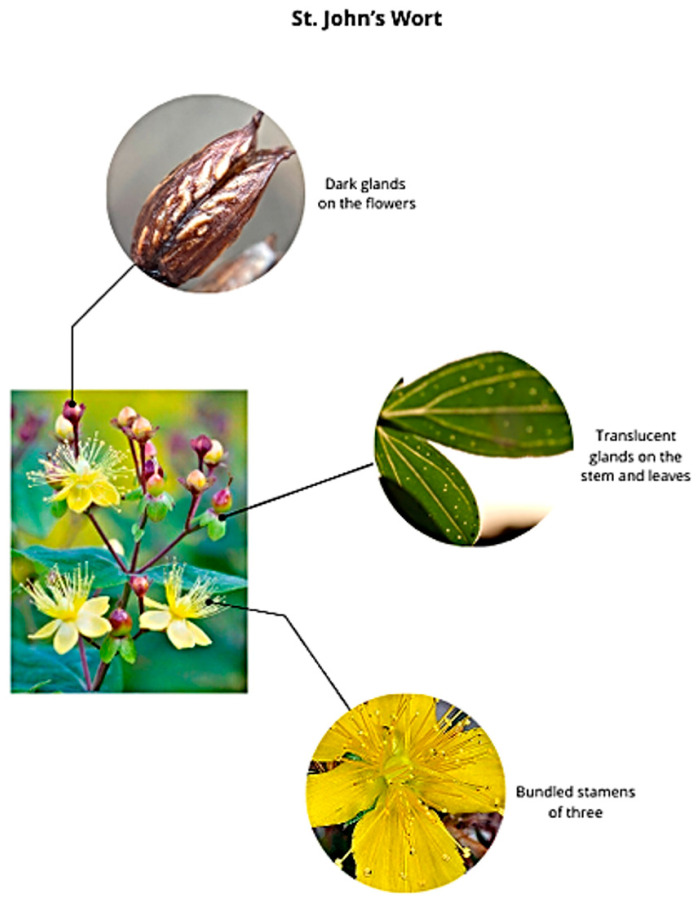
The main parts of SJW.

*Hypericum perforatum* L. is the most widespread species of the Hypericaceae family, primarily found mainly in the temperate regions of Eurasia and Northwest Africa [12]. All plants in the genus Hypericum have clustered stamens and glands, but what makes SJW stand out are the bundled stamens in groups of three, the translucent glands on the stem and leaves, and the dark glands on its flowers [12] (Figure 1).

### 3.1. Active Compounds of St. John’s Wort

The major active constituents are considered to be HPF (a prenylated phloroglucinol) and HYP (a naphthodianthrone), although other biologically active constituents, such as flavonoids and tannins, are also present [13,14]. The constituents of SJW, compiled from several sources, are presented below.

#### 3.1.1. Anthraquinone Derivatives (Naphthodianthrones)

Fresh material contains the biosynthetic precursors of HYP and pseudohypericin, specifically protohypericin and protopseudohypericin, as well as HYP, pseudohypericin, and isohypericin [15]. The presence of cyclopseudohypericin is also noted. The term ‘total hypericins’ refers to the hypericin content, which is approximately 0.1% to 0.15%, and is thought to include both hypericin and pseudohypericin [15].

#### 3.1.2. Flavonoids

Flavonols (quercetin, kaempferol); flavones (luteolin); glycosides (hyperoside, isoquercitrin, quercitrin, and rutin); biflavonoids, such as the flavone biapigenin and its derivative amentoflavone; and catechins, which are flavonoids frequently linked to condensed tannins, are all present. According to reports, the concentrations of rutin, hyperoside, and isoquercitrin are 1.6%, 0.9%, and 0.3%, respectively. Quercetin demonstrates powerful antioxidant properties, helping to neutralize free radicals and thereby reduce oxidative stress. Its therapeutic efficacy is notably increased through the synergistic effects of other compounds in the plant, such as HYP and HPF. On the other hand, rutin was found to be less potent than quercetin, possibly due to its lower bioavailability. Biapigenin protects neurons from excitotoxicity and mitochondrial dysfunction by reducing calcium accumulation while also exhibiting antioxidant activity that inhibits lipid peroxidation. Kaempferol appears to have a similar action as well [16,17].

#### 3.1.3. Prenylated Phloroglucinols

HPF (2.0% to 4.5%) and adhyperforin (0.2% to 1.9%) are oxygenated analogs of HPF.

#### 3.1.4. Tannins (8–9%)

They are among the most significant secondary metabolites of *H. perforatum*, exhibiting notable antimicrobial, anti-inflammatory, and antioxidant properties. These compounds contribute to the plant’s overall pharmacological profile by supporting its efficacy in combating infections and inflammation, as well as promoting wound healing [16,17]. The type is not specified. However, proanthocyanidins (condensed type) have been reported.

#### 3.1.5. Other Phenols

Caffeic, chlorogenic, p-coumaric, ferulic, p-hydroxybenzoic, and vanillic acids.

#### 3.1.6. Volatile Oils (0.05–0.9%)

Although present in lower concentrations and having less pronounced effects compared to the primary compounds like HYP and HPF, essential oils are believed to play a role in the plant’s mild sedative and antimicrobial properties. These oils include monoterpenes such as limonene, which offers antioxidant and anti-inflammatory benefits, and pinene, which enhances the plant’s aroma and may also provide anti-inflammatory, bronchodilator, and antimicrobial effects. Additionally, the oils contain sesquiterpenes, particularly caryophyllene, which imparts a spicy, woody fragrance and is thought to have anti-inflammatory and analgesic properties. The main component of the essential oils is methyl-2-octane (a saturated hydrocarbon, making up at least 30%), along with other compounds like nonane, traces of methyl-2-decane, n-undecane, alpha-terpineol, geraniol, and small quantities of myrcene and humulene [16,17].

#### 3.1.7. Other Constituents

Alcohols (C24, C26, C28), straight-chain saturated hydrocarbons (C16, C30), pectin, β-sitosterol, carotenoids, choline, nicotinamide, and acids (isovaleric, nicotinic, myristic, palmitic, and stearic) [13,14] are also present.

HYP is one of the most important and extensively studied compounds of the plant and is considered the most potent natural photosensitizer, with features such as strong long-wavelength absorption, minimal dark toxicity, certain tumor selectivity, and a high clearance rate from the host body [16,17]. SJW plays an important role in gene regulation [16,17]. Specifically, HPF participates in energy metabolism by affecting genes involved in the transport of exogenous and endogenous compounds. It also participates in intracellular calcium regulation, cell proliferation, and apoptosis [18].

### 3.2. Side Effects of St. John’s Wort

SJW is considered a well-tolerated drug, with rare and mild side effects that are transient [16,17]. The most frequently reported symptoms include gastrointestinal disturbances, disturbances of consciousness, skin problems, dry mouth, and allergies. However, these side effects occur in only 1–3% of patients taking the drug. In very rare cases, symptoms of phototoxicity, such as skin reactions, alopecia, neuropathy, and mania, have been reported.

Various studies also suggest that its concurrent administration with many other drugs may involve certain risks [16,17]. This was explained in a study by Mueller et al. [18], which showed that the content of ingredients in a herbal medicinal product determines the existence and magnitude of any potential interactions with other drugs. In fact, the study found that the necessary information about the analytical components of herbal preparations is often lacking or insufficiently reported, even in a large number of published studies related to herbal medicines [18].

## 4. Inflammation and Diabetes Mellitus

Inflammation is the immune system’s reaction to pathogens, damaged cells, toxins, or radiation. It works by eliminating harmful stimuli and initiating the healing process [19,20,21]. Thus, inflammation is an essential defense mechanism for maintaining good health. Numerous cell types form a highly coordinated network during the inflammatory response. Local reactions to infection and tissue damage are mediated by activated monocytes, macrophages, and other cells. Damaged epithelial and endothelial cells at injury sites release chemokines and growth factors that attract neutrophils and monocytes, as well as other factors that trigger the inflammatory cascade. Neutrophils are the first to be drawn to the injury site, followed by monocytes, lymphocytes (including T, B, and natural killer cells [NK]), and mast cells. Monocytes are recruited into injured tissues by chemotaxis and have the ability to differentiate into dendritic cells and macrophages. Numerous illnesses, such as diabetes, atherosclerosis, cancer, asthma, autoimmune and degenerative diseases, and chronic inflammatory diseases, are linked to changes in inflammation-mediated immune cells.

Macrophages play a key role in the body’s response to acute inflammation, but they also often contribute significantly to the long-term development of chronic diseases. The pathophysiology of DM and insulin resistance is strongly associated with obesity, a chronic, low-grade inflammatory disease. The liver, muscles, adipose tissue, hypothalamus, and pancreas are among the tissues that exhibit inflammatory changes induced by macrophages [19,20,21]. There are two distinct macrophage phenotypes that arise in response to changes in the local environment [22,23]. M1 macrophages, or classically activated macrophages, are responsible for releasing pro-inflammatory cytokines and chemokines, thereby promoting the development of local and systemic insulin resistance. The second phenotype, M2 macrophages, or alternatively activated macrophages, are responsible for producing primarily growth factors [22,23].

Partial or even complete loss of pancreatic β cells in DM appears to be significantly associated with increased levels of circulating pro-inflammatory cytokines, such as interleukin 1β (IL-1β), which regulates and enhances various immune responses, tumor necrosis factor (TNF-α), and interferon-gamma (IFN-γ), as well as free fatty acids, which disturb the homeostasis of the endoplasmic reticulum (ER) and lead to increased levels of stress. Islet death in DM significantly affects ER stress and the subsequent unfolded protein response, which, under normal conditions, plays an important role in β-cell survival [24,25]. Thus, combined ER stress, inflammation, and mitochondrial dysfunction are some of the main factors contributing to β-cell apoptosis in DM [24,25].

Cytokines such as IL-1β, IFN-γ, and TNF-α target pancreatic cells and activate signal transducer and activator of transcription-1 (STAT-1), as well as nuclear factor-κB (NF-κB) by phosphorylating them, thereby acting as strong transcription factors. In parallel, activation of the MAPK pathway may also contribute to the enhancement of inflammatory and apoptotic effects in pancreatic β cells [26]. MAPK is a serine/threonine protein complex composed of α, β, and γ subunits, which have two or three different isoforms. It appears to function depending on intracellular energy levels in response to various conditions such as hypoxia, glucose deprivation, mitochondrial damage, etc. The main role of this kinase is to maintain tissue homogeneity under conditions of metabolic stress, which is why it is considered a major therapeutic target for obesity and T2DM [27]. Results about oxidative stress and inflammation in diabetes mellitus in Table 1.

## 5. Oxidative Stress and Diabetes Mellitus

Blood glucose levels are not properly controlled in T1DM and T2DM, resulting in prolonged elevated levels [31,32]. Chronic hyperglycemia, a persistently elevated blood sugar level, is a characteristic feature of diabetes and a major contributor to the various complications associated with the condition. It is widely acknowledged that oxidative stress (OS) plays a critical role in the development and progression of diabetes, even though many aspects of the physiological mechanisms underlying this condition remain unknown [31,32].

When the generation and elimination of reactive oxygen species (ROS) are out of balance, OS results lead to the buildup of oxidants. ROS include peroxynitrite (ONOO^−^), superoxide anion (O_2_^•−^), and hydroxyl radical (^•^OH) [28,29]. Non-radical oxygen derivatives, such as hydrogen peroxide (H_2_O_2_), are also classified as ROS because they readily generate free radicals. ROS have important biological functions and are produced during regular cellular metabolism. Although they are essential for life, their high reactivity can damage macromolecules like proteins, lipids, and nucleic acids. As a result, cells activate defense systems to control the generation of ROS and prevent oxidative damage. Enzymes that neutralize excess ROS, such as glutathione peroxidases, peroxiredoxins, thioredoxins, catalase, and superoxide dismutases (SODs), form the main line of defense against ROS [28,29].

Mitochondria, organelles necessary for energy metabolism, play a crucial role in oxidative phosphorylation, which produces ATP. In this process, the electron transport chain (ETC) oxidizes nicotinamide adenine dinucleotide (NADH) and flavin adenine dinucleotide (FADH_2_), which are products of nutrient oxidation. This generates ATP, ROS, and, most importantly, superoxide (O_2_^•−^). As a result, mitochondria are the primary source of ROS within cells and contain antioxidant enzymes that help maintain cellular redox balance. The presence of a specific type of mitochondrial superoxide dismutase (MnSOD) that neutralizes O_2_^•−^ underscores the importance of mitochondria in both ROS production and their regulation to preserve homeostasis.

Glucose serves as the primary energy source for the ETC, where it is converted into NADH and FADH_2_ [30,33]. Therefore, it is not surprising that ROS are implicated in the physiopathology of diabetes. Evidence suggests that antioxidant enzymes are disrupted in individuals with T2DM, and numerous studies have reported a general state of OS in individuals with DM. Additionally, mitochondrial ETC dysfunction has been linked to diabetes, particularly in cases of mitochondrial diseases [30,33].

**Figure 2:**
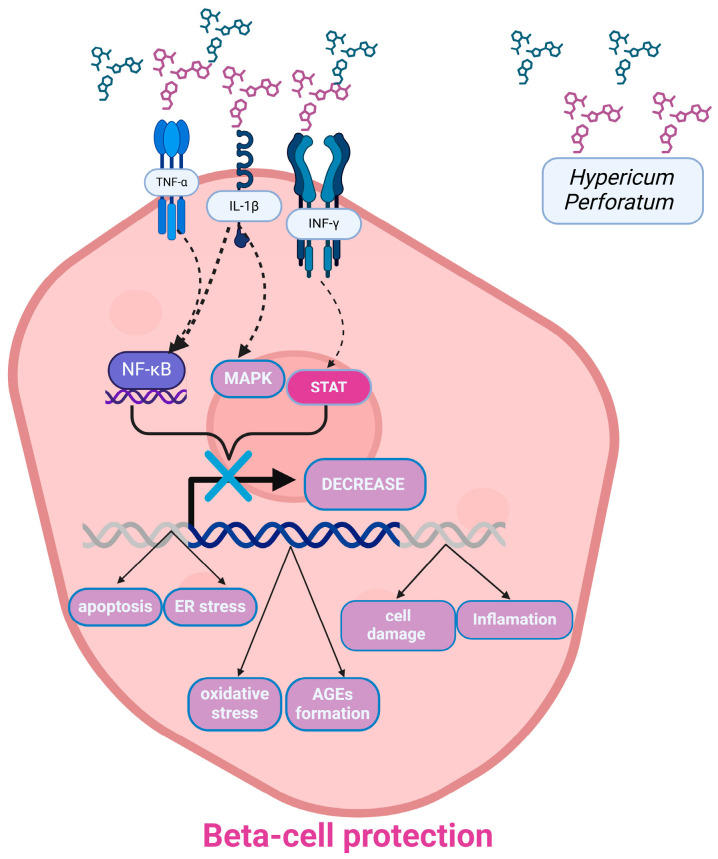
Protective mechanisms of St. John’s Wort extracts against cytokine-induced signaling Pathways in pancreatic β cells. TNF-α, tumor necrosis factor α; IL-1β, interleukin-1β; INF-γ, interferon-γ; SJW, St. John’s Wort; HPF, hyperforin; NF-κB, nuclear factor kappa-light-chain-enhancer of activated B cells; MAPK, mitogen-activated protein kinase; STAT, signal transducer and activator of transcription; ER, endoplasmic reticulum; AGEs, advanced glycation end-products. Created with www.BioRender.com by Anastasiou IA.

Proteins required for the ETC and ATP synthesis are encoded by the mitochondrial genome, and mutations in this genome have been linked to an increased risk of diabetes [19,34]. For instance, a recent study on individuals with mitochondrial disease found that endocrine disorders, such as DM, were more common in these patients. Increased OS has also been associated with mitochondrial diseases in the literature. Given the evidence linking T2DM and mitochondrial OS, it has been proposed that mitochondria play a critical role in the onset of diabetes and the subsequent production of ROS, which profoundly impacts the course of the disease [19,34] (Figure 2).

## 6. In Vitro Studies

Researchers found that SJW extracts inhibited 3T3-L1 cells’ ability to produce fat and also showed that they prevented mature fat cells from absorbing insulin-sensitive glucose [35]. 3T3-L1 is a fibroblast cell line isolated from a mouse embryo and is used to study the basic cellular mechanisms associated with DM and obesity. The researchers further described the effects of SJW on insulin action in both human and murine fat cells in these follow-up studies. SJW has also been shown to reduce insulin-sensitive glucose uptake in human adipocytes. Additionally, SJW prevents the tyrosine phosphorylation of IRS-1 in fat cells from both humans and mice. The capacity of HI and HF, which are isolated from SJW, to alter adipocyte maturation and insulin action has been investigated. HI and/or HF do not mediate the significant effects of SJW on adipogenesis, IRS-1 activation, or insulin-stimulated glucose uptake, according to this new study. However, the study suggests that SJW extracts may significantly increase insulin resistance in mature fat cells and limit preadipocyte differentiation, which may lead to adipocyte-related diseases [35].

Novelli et al. performed a series of studies attempting to demonstrate whether SJW and HPF, in particular, can modulate the action of cytokines in β cells, offering protection against cytotoxicity [26,36]. In the 2016 study, glucose-dependent rat insulinoma cell line INS-1E cells were used, and a standardized HPLC-titrated hydroalcoholic extract of SJW containing 25.7% HPF was applied, along with the cytokines IFN-γ, IL-1β, and TNF-α, which are released within the islets during the autoimmune insulitis process. Their results showed that the SJW extract, through HPF, can significantly reduce or even completely inhibit cytokine-induced activation and phosphorylation of STAT-1, NF-kB, and MAPK in pancreatic β cells while being unable to regulate target gene expression or prevent imbalances between pro- and anti-apoptotic genes [26]. In a corresponding study conducted in 2019, they investigated the previously unknown time course of the inhibitory effect of the SJW extract on STAT-1 and DNA binding in pancreatic β cells, aiming to gain a deeper understanding of how long it can maintain its action in a potential future pharmacological application of SJW or HPF treatment. INS-1E cells were used, and a hydroalcoholic extract of SJW containing 25.7% HPF and 0.12% HYP (by HPLC) was applied. Various experiments were conducted in which INS-1E cells were exposed to different concentrations of SJW or HPF for varying time intervals before and after the addition of a cytokine mixture. SJW extract at 5 and 2 μg/mL completely prevented the potent activation of STAT-1 and retained approximately 90% and 70–80%, respectively, of its inhibitory effect when added simultaneously with the cytokines or 15 min later. Similarly, HPF at 1 and 0.5 μM almost abolished STAT-1 activation when added 1 h before cytokines and was also very effective when added simultaneously or 15 min after cytokine addition. Therefore, it was clearly shown that the optimal pre-incubation period for achieving the maximum inhibitory effect was 1 or 2 h, while a 30 min pre-incubation period was sufficient for a marked reduction in STAT-1. Furthermore, if SJW or HPF were added simultaneously with the cytokine mixture, they could inhibit STAT-1 activation by about 90% at the highest dose. Additionally, it was shown that preexposure of INS-1 cells for 2 h to 2 or 5 μg/mL SJW significantly inhibited STAT-1 activation even after removal from the incubation medium [26,36].

In another study, among various plant compounds, they chose HYP as the most effective one against cell apoptosis and endothelial damage resulting from methylglyoxal (MGO) application [37]. MGO is a reactive compound primarily derived from the glycolytic pathway, resulting from the nonenzymatic degradation of the intermediate products dihydroxyacetone phosphate (DHAP) and glyceraldehyde-3-phosphate (G3P). It can form in the presence of hyperglycemia, inflammation, or hypoxia and is highly associated with the development of severe vascular complications in T2DM. Given its direct relation with insulin resistance, MGO has been linked to obesity, cardiovascular disease, cancer, and other neurodegenerative diseases. Thus, combating its presence is crucial, which can be achieved enzymatically through the glyoxalase system that converts MGO into D-lactate [38]. Researchers used cell cultures of human umbilical vein endothelial cells (HUVECs), which were incubated in HYP concentrations from 0.1 µM to 10 µM for 1 h, followed by a 24 h treatment with 400 µM MGO. The results showed that HUVECs exposed to MGO had an increased number of apoptotic cells and significant morphological changes. In contrast, with the effect of HYP, these changed, and the percentage of cell destruction decreased. To further study cell apoptosis, they investigated different proteins from the MAP-Kinase cascade related to mitochondrial function and oxidative damage. After HYP treatment, MAPK pathway activation was reduced, as were the higher ROS levels induced by 400 μM MGO, confirming the potential antioxidant activity of HYP. Regarding cell apoptosis, HYP concentrations exceeding 1 µM did not appear to have any significant effect. However, in the case of AGEs, there was an increase after treatment with BSA-MGO, whereas only 10 µM (and not 1 μM) of HYP appeared to significantly reduce the levels of AGEs. Nevertheless, aminoguanidine, used as a positive control, was more potent than HYP. HYP, a red naphthodianthrone derived from anthraquinones, appears to have inhibitory abilities against AGE production. Thus, through the inhibition of these harmful products and OS, as well as apoptotic regulation, HYP—the main component of *Hypericum perforatum* L.—can prevent the cytotoxicity caused by MGO and, by extension, be used as a potential protective agent against diabetic vascular complications [37].

In another study, the antidiabetic activity of SJW extract was investigated by determining its ability to inhibit α-amylase and α-glucosidase enzymes, using extracts from the roots (RO), non-flowering shoots (NFS), and flower shoots (FS) of the plant [38]. The results showed a dose-dependent inhibitory effect between NFS and FS, while more specifically, for α-amylase, one dose of 250 μg/mL RO and FS had a slightly higher inhibitory capacity (27%) than NFS (21.4%). Similarly, one dose of 150 μg/mL RO was significantly more potent (26.5%) than NFS and FS (17%), while one dose of 50 μg/mL RO was stronger (18.1%) compared to FS (10%) and NFS (6%). However, all tested extracts had lower inhibitory capacity than acarbose (79–89%), which was used as a specific enzyme inhibitor. Regarding a-glucosidase, one dose of 250 μg/mL RO showed a significantly higher inhibitory capacity (94,1%) compared to NFS (67.2%) and FS (70.4%), while one dose of 150 μg/mL RO was stronger (92.6%) than NFS (45.7%) and FS (61.5%). Finally, one dose of 50 μg/mL RO was stronger (88.9%) than FS (51.5%) and NFS (19.2%). The results were comparable to acarbose (96%) as a specific enzyme inhibitor, except for the 50 μg/mL dose. In conclusion, the RO extracts exhibited the strongest inhibitory activity against both enzymes, likely due to the xanthones and oligomeric procyanidins contained in the plant extracts, making them beneficial in the treatment and management of hyperglycemia [38].

In the study by Liang et al. [39], INS-1 cells were incubated in different concentrations of HYP (20 nM, 200 nM, 2000 nM) in culture media containing 33 mM glucose for 72 h, or 200 μM RA for 24 h. The protective effects of HYP on cell viability under glucotoxicity and lipotoxicity were assessed by the 3-(4,5-dimethyl-2-thiazolyl)-2,5-diphenyl-2-H-tetrazolium bromide (MTT) assay. The results showed that high glucose (33 mM) significantly reduced INS-1 cell viability by inducing apoptosis compared to normal control cells cultured in an 11 mM glucose medium. HYP was able to dose-dependently improve cell viability under these glucotoxic conditions. HYP also protected INS-1 cells from apoptosis due to induced lipotoxicity, indicating that it is a substance with the potential to reduce overall rates of cell damage while contributing to the inhibition of NO production and restoration of iNOS expression to normal levels [39]. Results from in vitro studies are presented in Table 2.

## 7. In Vivo Studies

Previous studies examining the anti-inflammatory effects of SJW extract have been reported in obese diabetic animal models. Specifically, it has been observed that the HPF and HYP contained in balsamroot extract prevent the early death of β-pancreatic cells and inhibit inflammatory signaling pathways in diabetic mice by inhibiting cytokines and pro-inflammatory factors such as NF-κB, STAT-1, IL-1β, TNF-α, and prostaglandins [40,41]. More specifically, in vivo studies have shown that SJW extract inhibits the action of 5-lipoxygenase (5-LO) in the generation of leukotrienes (A4, B4, C4, D4, E4) by preventing the phosphorylation of Ser271 and Ser663, thereby preventing the translocation of 5-LO to the nucleus for leukotriene production [42]. Moreover, an inability to catalyze 5-hydroperoxyacetate tetraenoic acid at leukotriene A4 by 5-LO was observed, blocking the formation of additional pro-inflammatory factors, including leukotrienes B4 and C4 [42,43]. At the same time, SJW was found to inhibit the formation of prostaglandin E2 (PGE-2), thereby reducing both acute and chronic inflammation [44,45]. It was revealed that the included HPF inhibits the enzymatic conversion of prostaglandin H2 (PGH-2) to PGE-2 via microsomal synthesis of prostaglandin E-1 [44,45].

Furthermore, according to Chen et al. [46], HPF was shown to contribute to the management of obesity associated with DM in animal models by increasing thermogenesis in adipose tissue. The activation of thermogenesis by HPF appeared to occur through a similar mechanism to the response to cold or food consumption. The results of this activation included a reduction in fat storage in the grey and white adipose tissue of the rats, preservation of lean body mass, and a reduction in total body weight for at least 3 weeks after the intervention. Some of the mechanisms responsible for these effects were an increase in the MAPK-induced thermogenic uncoupling protein-1 (UCP1) and mitochondrial number in the cells of the mice [46]. The UCP1 gene is a determinant in heat production, primarily in grey adipose tissue, regulated by the sympathetic nervous system. It directs energy production by uncoupling oxidative phosphorylation, which pushes protons in the respiratory chain into heat production. As for MAPK, it is an important protein that helps with cold perception and the initiation of thermogenesis in adipocytes, protecting cellular mitochondria and preventing the onset of diseases such as insulin resistance or metabolic fatigue [47,48,49].

Moreover, Galeotti et al. [50] described that the seed extract of the SJW plant significantly suppressed neuropathic pain in rats with diabetic neuropathy, highlighting the analgesic effects of the contained HPF and HYP [50]. Similar conclusions were reached by Galeotti et al. [51], who pointed out that even low doses of HPF in diabetic animal models can relieve painful syndromes with high safety, preventing adverse interactions with other herbs or drugs and reducing the need for opioids [51]. Additionally, based on the study by Abd El Motteleb and Abd El Aleem [52], HPF was shown to improve diabetic nephropathy in experimental animals by increasing antioxidant factors, suppressing inflammatory cytokines, reducing fibrotic effects, and lowering blood glucose. Specifically, in this study, hyperic acid was observed to reduce hyperglycemia in experimental animals, which is one of the main triggers of diabetic nephropathy in mice, through the induction of the NF-кB factor and the oxygen and nitrogen free radical (ROS and NOS) pathway, respectively. Due to the inactivation of NF-кB by SJW, the induction of inflammatory and apoptotic substances, such as TNF-α, IL-1β, and cyclooxygenase II, was interrupted, while the formation and secretion of adhesion molecules, such as monocyte chemoattractant protein-1 (MCP-1), which aggravates renal damage, was stopped. At the same time, the inactivation of protein kinase C (PKC) and C-reactive protein (CRP) in the serum of diabetic mice after hyperic acid administration was documented due to suppression of marked hyperglycemia [52].

Rafailovoska et al. (2023) investigated the effect of hyperphosphine in diabetic rats and found that the administration of this substance in an amount of 200 mg/kg was able to normalize blood glucose levels [53]. Some of the mechanisms contributing to this effect were the reduction of liver enzymes involved in glucose metabolism, the increase in insulin levels, and the reduction of insulin resistance [53]. Similar conclusions were reached by previous studies, emphasizing the insulinotropic effect of SJW extract [39,54]. The insulinotropic effect of the extract is probably due to the reduction of poly (ADP-ribose) polymerase (PARP) activity in the pancreas, which reduces the apoptosis of pancreatic β cells and improves insulin production and secretion [53]. It seems that PARP, in oxidative or hyperglycemic environments, contributes to the repair of genetic material by mediating the destruction of insulin-producing cells [53,55,56]. On the other hand, it should be mentioned that in some diabetic mice, the activity of the PARP enzyme increased after the use of SJW, likely due to the antioxidant activity of the extract and its ability to destroy oxygen free radicals [16,53].

In the study by Rafailovoska et al. [53], improvements in carbohydrate metabolism were observed in diabetic rats. Specifically, they noted a rise in gluconeogenesis through an increase in glucose 6-phosphate dehydrogenase and a reduction in glycolysis due to a decrease in glucose delivery to the liver and MAPK signaling. At the same time, there was a decrease in the pentose phosphate pathway due to a reduction in glucose 6-phosphate dehydrogenase in other metabolic pathways [57,58,59]. These processes indirectly contribute to maintaining glucose levels and storing it as glycogen in the liver, thereby normalizing sugar levels in the bloodstream. The study showed no effect on glycogen phosphorylase, meaning the glycogenolysis pathway was not significantly altered in the bodies of diabetic rats treated with HPF [52]. Moreover, previous studies have reported positive effects of SJW in the management of prediabetes, demonstrating improvements in carbohydrate metabolism in insulin-resistant mice through inhibition of protein tyrosine phosphatase [41,51].

In another study on diabetic mice, SJW extract was shown to inhibit the action of paracetamol in increasing the release of the enzyme myeloperoxidase from the azurophilic granules, where it is normally stored, reducing the levels of oxidized glutathione in the serum of experimental animals and preventing liver toxicity associated with paracetamol ingestion. It also prevented the mobilization of cytokines and inflammatory cells [60]. Similar results were obtained by Uslusoy et al. (2019), who, studying rats with neuropathy, showed that hyperic acid extract inhibits the depletion of cellular antioxidant components and reduces the overexpression of apoptosis and necrosis enzymes of β-pancreatic cells [61]. Furthermore, in another in vivo study, SJW was observed to inhibit the occurrence of edema, ischemia, and pancreatitis in mice receiving short-term cerulean while also contributing to increased survival [61]. Results from in vivo studies are presented in Table 3.

## 8. Human Studies

Regarding human studies, based on case reports, the use of HPF as part of plant extracts appeared to contribute to wound healing in individuals with diabetic foot ulcers, promoting faster skin resurfacing [62,63]. A study by Iabichella et al. reported on a 72-year-old patient with T2DM for 30 years, cardiomyopathy, and arteriopathy in the lower limbs, most pronounced in the left leg [62]. From April 2011, when the patient was first followed up, to July 2011, the patient showed improvement in glycosylated hemoglobin (HbA1c), with a 0.4% drop (from 8.0% to 7.6%), but worsening of microangiopathy in the right lower limb, where a minor amputation was performed in August 2011. Arteriopathy in the left leg was stable during the aforementioned period but deteriorated severely in the following months, with the worsening of the pre-existing ulcer. Due to transportation difficulties, the patient and the doctors agreed to treat the ulcer with an extract containing HPF. After a few months of treatment, the ulcer improved, fibrogenic activity was reduced, and glycemic control improved with stabilization of HbA1c at 7.6–7.7% [62].

The following year, Iabichella’s team presented another case demonstrating improvement in diabetic wounds due to the use of an extract containing HPF [56]. They reported on a 67-year-old woman with obesity (Body Mass Index: 32 kg/m^2^) and T2DM for 25 years, who had a left lower limb ulcer and a long-standing symptomatic diabetic neuropathy. At baseline, the patient had poor glycemic control (fasting glucose was 271 mg/dL, and HbA1c was 14.2%). Despite the worsening of the ulcers on the leg, the patient refused amputation, so the extract was applied to the wounds. This contributed to the improvement of skin reconstruction, cleanliness of the area, and a reduction of fibrinolysis. Simultaneously, hyperglycemia improved significantly, with a continuous decrease in HbA1c (first to 10%, then to 8%, and 6%, respectively) and a reduction in fasting glucose to 130 mg/dL by the end of the intervention. The diabetic ulcers continued to improve, and the patient was able to walk on her own again [63]. The enhancing effect of HPF on the healing of diabetic wounds and DM in general has also been documented in in vivo studies [64,65].

Twenty healthy male participants in an open cross-over study were given 1 g of metformin twice a day for one week, both with and without 21 days of concurrent and prior St. John’s Wort treatment [66]. A two-hour oral glucose tolerance test was conducted in addition to determining the pharmacokinetics of metformin. According to their findings, SJW reduced metformin’s renal clearance while having no effect on any other pharmacokinetic parameters of metformin. The addition of SJW decreased the area under the glucose concentration–time curve [702 (95% CI, 643–761) vs. 629 min·mmol/L (95% CI, 568–690), *p* = 0.003]. A statistically significant increase in the acute insulin response was the cause of this effect. In healthy male subjects taking metformin, SJW increases insulin secretion, thereby improving glucose tolerance independent of insulin sensitivity [66].

## 9. Discussion

*H. perforatum* is reported to contain several phytochemical constituents, such as rutin and flavonoids, including quercetin and isoquercetin. It is well known that natural products derived from plants, which are rich in bioactive compounds with scientifically proven efficacy—like the SJW extract discussed here for its potent anti-inflammatory action—offer a valuable substitute or complement to traditional pharmaceutical interventions. These products are considered safer and more suitable for long-term treatment, making them especially appropriate for a preventive approach. Adequate evidence from both in vitro and in vivo research indicates that *H. perforatum* has strong antihyperglycemic properties and few side effects. Some components of SJW may influence glucose metabolism or insulin sensitivity, but it is important to note that more research is needed to draw definitive conclusions.

With the possible exception of patients who are depressed, it must be acknowledged that SJW’s and its component HPF’s interference with drug metabolism is likely a major factor in the supplement’s limited use in clinical trials to date. The scientific community’s continued lack of knowledge about SJW’s extraordinary anti-inflammatory properties could be another factor. In any event, the insufficient clinical research that has been carried out thus far represents a temporary limitation on the use of SJW’s characteristics, which should be addressed shortly. Another intriguing viewpoint is the discovery that several HPF analogs retain the ability to activate the transient receptor potential channel 6 (TRPC6), which is probably involved in the mechanism of HPF’s antidepressant action, but are unable to induce pregnane X receptor activation and drug metabolism [9,67]. More research is required to determine whether these analogs retain the anti-inflammatory and antidiabetic properties of HPF.

## Figures and Tables

**Table 1:** Studies about oxidative stress and inflammation in diabetes mellitus.

Substance/Pathway	Main Role	Findings	Author and Reference
Mitochondrial DNA (mtDNA)	OS and inflammation balance	Mitochondrial dysfunction from mtDNA mutations contributes to diabetes by disrupting energy metabolism, insulin resistance, and β-cell function. Mutations affecting oxidative phosphorylation and ATP production increase OS and inflammation, worsening diabetic conditions. These mutations are linked to insulin resistance in T2DM by disturbing energy balance and glucose uptake. Targeting mtDNA dysfunction may offer potential therapies, with interventions aimed at repairing mtDNA or improving mitochondrial function. Further research is needed to develop mitochondria-targeted treatments for diabetes.	Al-Ghamdi et al. [19]
IRE1 (Inositol-requiring enzyme 1) XBP1 (X-box binding protein 1)	Managing ER stress	Cytokine exposure reduces adaptive UPR gene expression and XBP1 splicing while increasing pro-apoptotic genes like CHOP and Trib3. Inhibition of XBP1 worsens β-cell death, highlighting its role in cell survival. JNK activation shifts the UPR from adaptive to apoptotic, and JNK inhibition protects β cells. The study suggests that modulating the XBP1:CHOP balance and JNK activity could offer therapeutic strategies for diabetes.	Chan et al. [24]
Multimeric Ca^2+^ -binding glycoprotein thrombospondin 1 (THBS1)	Cytoprotective and anti-apoptotic factor	Protects β cells from cytokine and ER stress-induced apoptosis by upregulating mesencephalic astrocyte-derived neurotrophic factor (MANF). THBS1 sustains MANF expression, which is crucial for β-cell survival, and its protective effect is dependent on MANF. Silencing MANF abrogates THBS1’s cytoprotective role, with BIM acting as a key mediator of apoptosis. The THBS1–MANF pathway provides partial protection in human and mouse β cells but not in rats. These findings highlight the potential of targeting the THBS1–MANF pathway as a therapeutic strategy for β-cell protection in diabetes.	Cunha et al. [25]
SJW HPF	Anti- inflammatory	SJW and HPF effectively restore glucose-stimulated insulin secretion, mitigate pro-inflammatory gene expression and nitric oxide (NO) production, and provide protection against apoptosis and necrosis. Notably, SJW demonstrates superior efficacy, improving the integrity of both mitochondria and insulin granules. These results indicate that the protective effects of these plant compounds on β cells are mediated through the modulation of STAT-1 and NF-kB signaling pathways.	Novelli et al. [26]
AMPK (AMP-activated protein kinase)	Anti-inflammatory and antioxidant	AMPK plays a crucial role in maintaining energy homeostasis by facilitating glucose uptake, fatty acid oxidation, and mitochondrial biogenesis while suppressing protein and lipid synthesis. Re-establishing AMPK activation has the potential to enhance glucose regulation, positioning it as a valuable target for diabetes therapy. Activators like metformin, berberine, and resveratrol increase AMPK activity, which in turn improves insulin sensitivity and lipid metabolism. This review underscores the therapeutic potential of AMPK activation in developing novel treatments for diabetes and other metabolic disorders.	Kim et al. [27]
Reactive oxygen species (ROS)	Pro- inflammatory and anti-inflammatory	Excessive production of ROS leads to oxidative stress, contributing to diseases like CVD, cancer, and neurodegeneration. Moderate ROS levels are essential for cellular signaling, immune defense, and metabolic regulation. ROS regulate processes involved in cell growth, survival, and inflammation. While antioxidants mitigate ROS damage, excessive use may impair their beneficial roles. Maintaining a balance between ROS production and antioxidant defense is crucial for developing therapies for oxidative stress-related diseases.	Pizzino et al. [28]
Antioxidants	Antioxidant	Antioxidants, including vitamins C and E, flavonoids, and other compounds, lower ROS levels, thereby safeguarding β cells from damage and maintaining their function and insulin secretion. These antioxidants neutralize ROS, promote cellular repair, and regulate inflammatory pathways. Preclinical studies demonstrate that antioxidants can mitigate β-cell dysfunction and enhance insulin secretion. However, additional research is required to identify the most effective antioxidants and fully elucidate their mechanisms of action.	Anastasiou et al. [29]
Mitochondrial electron transport chain (ETC)	Maintaining cellular energy balance	ETC is critical for oxidative phosphorylation, enabling ATP production through electron transfer from nutrients to oxygen. It also generates reactive oxygen species (ROS), which, in excess, can cause oxidative stress and cellular damage. Dysregulation of the ETC and increased ROS production are associated with neurodegenerative, cardiovascular, and metabolic diseases. Understanding the balance between ATP production and ROS generation is vital for therapeutic development.	Nolfi-Donegan et al. [30]

**Table 2:** The effects of *Hypericum perforatum* L. on cell lines.

In Vitro Studies/Cell Lines	SJW Extract	Results	Author and Reference
Rat and human IFN-γ, IL-β and TNF-α	HPF	HPF prevents cytokine-induced disruption of insulin secretion, downregulates cytokine-induced expression of pro-inflammatory genes, and inhibits nitrite production while generally protecting against cell death.	Novelli et al. [26]
Murine 3T3-L1 (fibroblast cell line), human adipocytes	SJW extracts	SJW extracts may contribute to adipocyte-related diseases by limiting differentiation of preadipocytes and significantly inducing insulin resistance in mature fat cells.	Richard et al. [35]
β-cell lines and isolated rat and human pancreatic islets	HPF	Orally administered SJW extract, containing appropriate amounts of HPF, could be tested in clinical trials in patients with COVID-19 as a well-tolerated anti-inflammatory agent with significant potential to prevent or limit the effects of cytokine storm.	Masiello et al. [36]
Human umbilical vein endothelial cells (HUVECs)	HYP	HYP rescued the morphological changes and appeared to prevent MGO-induced apoptosis. Pre-treatment with HYP, prior to MGO stimulation, reduced the activation of three MAPKs that lead to apoptosis. At 10 µM, it also significantly reduced the formation of AGEs.	Do MH et al. [37]
Properties of roots (RO), non-flower shoots (NFS), and flower shoots (FS)	SJW	The plant extracts demonstrated antibacterial and antifungal activity, as well as significant, dose-dependent inhibition of monoamine oxidase activity. The RO extracts exhibited the strongest inhibitory activity against α-amylase and α-glucosidase, a finding that suggests they are beneficial in the treatment and management of hyperglycemia.	Tusevski et al. [38]
INS-1 cells (rat insulinoma cell line)	HYP	HYP inhibits apoptosis and NO production in INS-1 cells under high glucose and lipid toxicity, protecting them and maintaining PDX1 expression and Erk activation. Prophylactic use of HYP ameliorates diabetic phenotypes, reduces β-cell loss, preserves islet mass, improves insulin expression, enhances pancreatic antioxidant capacity, and prevents islet β-cell apoptosis in HFHS-fed mice.	Liang et al. [39]

**Table 3:** The effects of *Hypericum perforatum* L. on animal models.

In Vivo Studies/ Animal Model	SJW Extract	Results	Author and Reference
8-week-old C57BL/6J mice -ob/ob mice high-fat diet (HFD)- induced obese (DIO) mice	HPF	HPF treatment in mice showed a strong and lasting anti-obesity effect. It slowed weight gain without altering food intake, reduced total fat mass, enhanced thermogenesis, and improved glucose homeostasis.	Chen et al. [46]
Male Sprague–Dawley albino rats	SJW seeds	The results demonstrated SJW’s ability to relieve neuropathic pain, confirming previous findings. It appears that HYP likely plays a prominent role in the extract’s analgesic activity.	Galeotti et al. [50]
Streptozotocin (STZ) and nicotinamide (NA) rat model	SJW extract	HPF ameliorates diabetic nephropathy by increasing antioxidant factors, suppressing inflammatory cytokines, reducing fibrotic effects, and lowering blood glucose.	Abd El Motteleb et al. [52]
Male Wistar albino rats	HH powder (Hyperici Herba)	The extract improves carbohydrate metabolism, enhances insulinotropic activity, and regulates endogenous glucose production. It inhibits hepatic gluconeogenesis in diabetic rats by ameliorating AMPK and PKCε changes in the liver of STZ-diabetic rats while also reducing PARP activity in the pancreas.	Rafailovska et al. [53]
Male Swiss mice	HPE	HPE inhibits paracetamol-induced lethality and hepatotoxicity through the inhibition of cytokine production, neutrophil recruitment, depletion of GSH, and antioxidant capacity in the liver.	Hohmann et al. [60]
Rats	HPL	HPL treatment reduced high levels of lipid peroxidation in muscles, brain, and red blood cells while increasing the peroxidase activity of GPx, α-tocopherol, and melatonin, leading to the prevention of inflammatory, oxidative, and apoptotic damage.	Uslusoy et al. [61]

## Data Availability

Not applicable.

## Abbreviations

5-LO	5-lipoxygenase
AGEs	advanced end glycation products
BCL6	B-cell lymphoma 6
CCL11	C motif chemokine 11
CRP	C-reactive protein
CVD	cardiovascular disease
DM	diabetes mellitus
ER	endoplasmic reticulum
ETC	electron transport chain
FADH_2_	flavin adenine dinucleotide
FS	flower shoots
HbA1c	glycosylated hemoglobin
HPF	hyperforin
HYP	hypericin
IFN-γ	interferon gamma
IL-1β	interleukin 1β
MAPK	mitogen-activated protein kinase
MGO	methylglyoxal
MnSOD	superoxide dismutase
MTT	3-(4,5-dimethyl-2-thiazolyl)-2,5-diphenyl-2-H-tetrazolium bromide
NADH	nicotinamide adenine dinucleotide
NFS	non-flowering shoots
NF-κB	nuclear factor-κB
NK	natural killer cells
O_2_•−	superoxide anion
PARP Poly	(ADP-ribose) polymerase
PGH-2	prostaglandin H2
PKC	protein kinase C
RO	roots
ROS	reactive oxygen species
SJW	St. John’s Wort
SODs	superoxide dismutases
STAT-1	signal transducer and activator of transcription-1
T1DM	type 1 DM
T2DM	type 2 DM
TNF-α	tumor necrosis factor
TRPC6	transient receptor potential channel 6
UCP1	uncoupling protein-1
